# The Economic Impact of Starting, Stopping, and Restarting an Antibiotic Stewardship Program: A 14-Year Experience

**DOI:** 10.3390/antibiotics2020256

**Published:** 2013-04-24

**Authors:** Mary A. Ullman, Garry L. Parlier, James Bryan Warren, Noe Mateo, Craig Harvey, Christopher J. Sullivan, Robert Bergsbaken, Isaac F. Mitropoulos, John A. Bosso, John C. Rotschafer

**Affiliations:** 1 Regions Hospital, St. Paul, MN 55101, USA; 2 Antibiotic Pharmacodynamic Research Institute, University of Minnesota College of Pharmacy, Minneapolis, MN 55455, USA; 3 University of Minnesota, Minneapolis, MN 55455, USA; 4 South Carolina College of Pharmacy, Charleston, SC 29208, USA; 5 Medical University of South Carolina College of Medicine, Charleston, SC 29208, USA

**Keywords:** antimicrobials, antibiotic costs, stewardship, resistance

## Abstract

Regions Hospital started a multidisciplinary antibiotic stewardship program (ASP) in 1998. The program effectively shut down from 2002–2004 as key personnel departed and was then restarted but without the dedicated pharmacist and infectious diseases physician. Purchasing data (in dollars or dollars/patient/day) unadjusted for inflation served as a surrogate marker of antibiotic consumption. These data were reviewed monthly, quarterly, and yearly along with antibiotic susceptibility patterns on a semi-annual basis. Segmented regression analysis was use to compare restricted antibiotic purchases for performance periods of 1998–2001 (construction), 2002–2004 (de-construction), and 2005–2011 (reconstruction). After 4 years (1998–2001) of operation, a number of key participants of the ASP departed. For the following three years (2002–2004) the intensity and focus of the program floundered. This trend was averted when the program was revitalized in early 2005. The construction, deconstruction, and reconstruction of our ASP provided a unique opportunity to statistically examine the financial impact of our ASP or lack thereof in the same institution. We demonstrate a significant economic impact during ASP deconstruction and reconstruction.

## 1. Introduction

Antibiotic stewardship is gaining universal acceptance as a valuable tool to limit bacterial antibiotic resistance, maximize clinical outcome with antibiotic therapy, minimize antibiotic induced adverse events, and to control cost. A recent policy statement by the Society for Healthcare Epidemiology of America (SHEA), the Infectious Diseases Society of America (IDSA), and the Pediatric Infectious Diseases Society (PIDS) recommends (1) antibiotic stewardship programs (ASP) be mandated through regulatory mechanisms; (2) antibiotic stewardship be extended into the ambulatory healthcare setting; (3) there should be formal education regarding antibiotic resistance and stewardship; (4) antibiotic usage data be collected and shared for inpatients and outpatients, and (5) antibiotic stewardship research is needed [1,2]. 

Regions Hospital (formerly Ramsey Medical Center) is a 455 bed, level-one trauma center, with ~120,000 patient days of care per year. Among the hospital units are multiple intensive care units, including a burn unit. In 1998, Regions Hospital started an ASP with many of the key elements identified later in the IDSA ASP position paper published in 2007 [2]. (Table 1) The program was initially staffed with 0.25 FTE infectious diseases (ID) physician and 1.0 FTE ID trained pharmacist. The program was interdisciplinary involving the pharmacy director, hospital administrator, microbiologist, information systems (IS) personnel, infection control, and the physician chair of the pharmacy and therapeutics committee. Initial ASP goals included optimizing clinical outcomes with antibiotic therapy, limitation of collateral damage due to overuse or inappropriate use of antibiotics, and cost savings.

Hospitals that have committed to an ASP experience a significant reduction in antibiotic cost following implementation [1,2,3,4,5]. We report here a somewhat unique situation where our ASP was constructed, de-constructed, and reconstructed over a period of 14 years and the resultant economic impact.

## 2. Experimental

### 2.1. Data Analysis

Monthly antibiotic purchasing for 1998 through 2010 were considered and analyzed in terms of total restricted and unrestricted antibiotics purchases, restricted antibiotics per patient/day, total antibiotics, total antibiotics per patient/day, and percent restricted to total antibiotics, with total purchase costs serving as a surrogate marker for utilization. Total patient days used census figures for adult inpatients including ICUs. Segmented regression analysis for interrupted time series [6] was used to determine significance for the difference in levels and slopes over time due to the two interruptions (changes in the nature/status of the stewardship program): (1) deconstruction (stop) of team in June 2001, and (2) a subsequent reconstruction (restart) of the core stewardship team in March 2005, Figure 2) The Durbin-Watson statistic was used to test for autocorrelation. If autocorrelation was detected, the parameter was corrected with Yule-Walker estimation. Significance was determined at the 0.05 level. 

**Table 1:** Recommendations by Infectious Diseases Society of America (IDSA) for Antimicrobial Stewardship Program [2].

Recommendations	Rank ^a^	Met by Program?
Multidisciplinary antimicrobial stewardship team with	A-III	Yes
Infectious Disease Physician		Yes
Clinical Pharmacist with ID training		Yes
Clinical Microbiologist		Yes
Information system specialist		Yes
Infection Control Professional		Yes
Hospital Epidemiologist		No
Collaboration between stewardship committee and	A-III	
Infection Control		Yes
Pharmacy and Therapeutics Committee		Yes
Support and collaboration of hospital administration, medical staff leadership ^b^	A-III	Yes
Function under quality control and patient safety	A-III	No
Negotiation with administration for adequate authority, compensation, expected outcomes	A-III	Yes
Prospective audit and feedback	A-I	Yes
Formulary restriction and guidelines	A-II	Yes ^c^
Education of staff	A-III	Yes
Guidelines and clinical pathways	A-I	Yes
Antimicrobial cycling	C-II	No
Antimicrobial order forms	B-II	Yes ^d^
Combination therapy	C-II	No
Streamlining/de-escalation of therapy	A-II	Yes
Dose optimization	A-II	Yes
IV-to-PO conversion	A-III	Yes
Health care information technology		
Electronic medical records	A-III	Yes ^e^
Computer physician order entry	B-II	Yes ^e^
Clinical decision support	B-II	Yes ^e^
Computer-based surveillance	B-II	No
Microbiology lab providing patient-specific culture and susceptibility data, surveillance of resistant organisms	A-III	Yes
Process measures	B-III	No
Outcome measures	B-III	Yes

^a^ As per the Infectious Diseases Society of America-United States Public Health Service grading system for ranking recommendations in clinical guidelines; ^b^ Includes pharmacy director, patient care committee, and medical executive committee; ^c^ Restriction of antibiotics was primarily utilized. Restricted antibiotics did not need pre-authorization, but directed the attention of antibiotic surveillance for patient evaluation. A handful of antibiotics were selected for the requirement of pre-authorization; ^d^ Antibiotic forms were utilized during some point over the 11 years, but it was hard to gain acceptance of the use of the forms. For this reason, they are no longer utilized; ^e^ The health care information technologies were recently implemented in the past two years of the antimicrobial stewardship program.

Data were missing or questionable for 4/156 monthly time periods. Thus we used the mean total restricted antibiotics for all other time periods to replace these four values of total restricted antibiotics. Other variables for restricted antibiotics per day and percent-restricted antibiotics were adjusted accordingly based on this modification.

## 3. Results

### 3.1. Stewardship Strategies

#### 3.1.1. Formulary Restriction and Preauthorization

The antibiotic formulary was divided into two groups of agents, restricted and non-restricted; restricted classification was driven primarily by cost, dosing difficulty and risk of toxicity (e.g., aminoglycosides), and/or agent novelty (Table 2). A patient receiving a restricted antibiotic initially did not require prior-authorization but these patients would be identified on the IS daily report and would be evaluated by the ASP team. Over time and with the introduction of new antibiotics, the “restricted” list expanded with some products requiring prior authorization by an infectious diseases physician.

**Table 2:** Example of antibiotic formulary classifications.

Restricted Antibiotics	Non-restricted Antibiotics
Amikacin	Acyclovir
Azithromycin (IV only)	Amphotericin B
Aztreonam	Ampicillin
Caspofungin	Ampicillin/sulbactam
Cefepime	Cefazolin
Cefotaxime	Cefotetan
Ceftazidime	Cefuroxime
Ceftriaxone	Chloramphenicol
Daptomycin ^a^	Clindamycin
Fluconazole	Doxycycline
Imipenem/Meropenem ^b^	Erythromycin
Levofloxacin	Gentamicin
Linezolid ^a^	Metronidazole
Lipid Amphotericin products	Nafcillin
Moxifloxacin	Penicillin
Piperacillin/tazobactam	Piperacillin
Quinupristin/dalfopristin ^a^	Trimethoprim/sulfamethoxazole
Tigecycline	
Tobramycin	
Vancomycin	
Voriconazole ^a^	

^a^ Must be approved by ID prior to use; ^b^ During the time period, the formulary carbapenem was switched from imipenem to meropenem.

#### 3.1.2. Prospective Audit with Intervention and Feedback

A list of patients receiving restricted antibiotics was generated daily by the hospital’s information systems department, and the ID physician, ID pharmacist, pharmacy, and chair of the P&T committee received copies to identify for possible interventions. 

In most cases, the pharmacy personnel and ID consultation personnel worked in tandem; however, if the pharmacist encountered a patient not also being seen via an ID consultant, the pharmacist contacted the prescribing physician and discussed possible treatment options. Unresolved cases between the pharmacist and prescriber were then referred to the ID consultant for further review and action.

#### 3.1.3. Monitoring of Process and Outcome Measures

In 1998, the only readily available and widely used metric to measure antibiotic consumption was antibiotic purchase data. As external comparisons of our performance were not possible, a decision was made to collect data over time and compare the internal performance of our ASP yearly. Over time, to maintain consistency in our internal comparisons, we have continued to use this metric. As a result, parameters of performance included total antibiotic purchases for the calendar year, total antibiotic cost per patient per day, restricted antibiotic cost per patient per day, percent of antibiotic dollars spent on restricted antibiotics, and the percent of antibiotic purchases as compared to total in patient drug purchases for the calendar year. 

The obvious drawback in using purchase data is that increases or decreases in annual antibiotic purchasing patterns are subject to the magnitude of inflation, better purchasing strategies, contract price changes, rebates, generic drug status, introduction of expensive new agents that are widely used, and now the purchasing and hording of antibiotics in short supply. However, this variability remained in play over the duration of the program. The clinical pharmacist and pharmacy director reviewed antibiotic use in the form of purchasing data monthly. These data were summarized and reviewed quarterly by the antibiotic subcommittee and passed on through the hospital committee structure. 

Annually and semiannually, the antibiotic subcommittee reviewed bacterial antibiotic susceptibility for Gram positive and Gram-negative pathogens as an alert for changing patterns of antibiotic susceptibility. The clinical microbiologist stratified these data by intensive care units and as comprehensive (hospital-wide) data. Based on antibiotic subcommittee decisions, any changes of 5% of more in susceptibility from year to year were considered to be significant and warranted further action as necessary. Antibiotic susceptibility data were reviewed for the first six months of the year and at the end of the 12 months. The clinical microbiologist also alerted the team to any unusual organisms or unusual resistance patterns between the 6 and 12 month periods.

A comprehensive yearly presentation of antibiotic purchase data and bacterial susceptibility data was prepared and shared with the infectious diseases physicians group, our hospital vice president, key physicians, the Pharmacy and Therapeutics Committee, the antibiotic subcommittee, and the pharmacy director. Minutes of the report are passed along to our Patient Care and Executive Committees.

### 3.2 Antimicrobial Purchases

**Figure 1:**
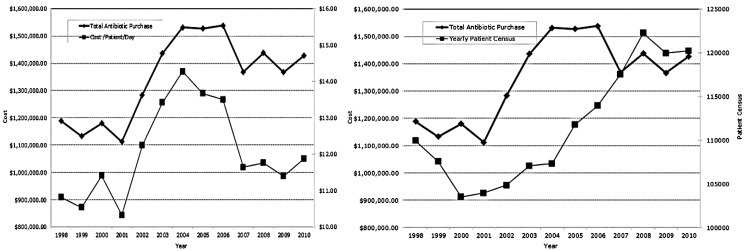
Total antibiotic purchases, Total antibiotic cost per patient per day, and annual census by year.

After initially establishing a baseline in 1998–2001, antibiotic costs (total restricted and unrestricted, total and restricted cost/patient/day) initially rose with the patient census 2002–2004 during ASP deconstruction and cost fell after reconstruction 2005–2010 despite continued increases in patient census (Figure 1). Restricted antibiotics (Table 2) consumed 55%–75% of the antibiotic dollar. Four or five antibiotics (levofloxacin, ceftriaxone, imipenem/meropenem, ertapenem, and vancomycin) depending on time period made up ~50% of the antibiotic budget. Over the fourteen years, antibiotic purchases represented <15% of the inpatient drug budget.

### 3.3. Effects of Deconstruction and Reconstruction of a Core Stewardship Team

Beginning in 2001, the antibiotic stewardship program experienced the loss of several key initial personnel on the ASP team, including the primary ID physician, the clinical microbiologist, and two clinical pharmacists who worked primarily with the stewardship program. With the departure of these individuals, the intensity and operation of the ASP floundered. Instead of a focused daily responsibility, these tasks were reassigned to the decentralized clinical pharmacists and made part of other daily clinical responsibilities. The program was revitalized in 2005 with a re-focusing of efforts on antimicrobial stewardship.

**Figure 2:**
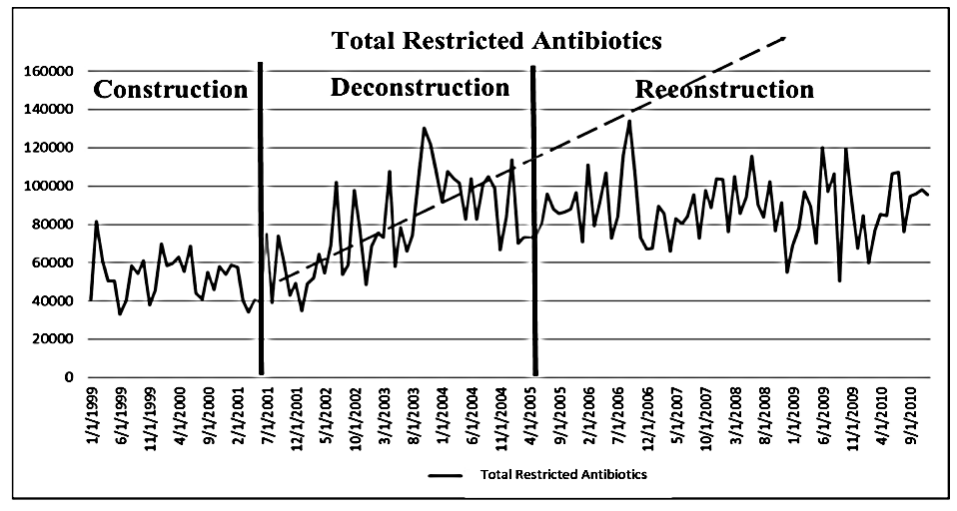
Segmented regression series analysis of total restricted antibiotic purchases by year during antibiotic stewardship program (ASP) construction (start), deconstruction (stop), and reconstruction (restart) See text for statistic description.

Using cost data for 2002, 2003 and 2004 and projecting future costs using the slope of that data into 2005 and beyond versus what actually happened after reconstruction clearly demonstrate the successful impact of restoring the program (Figure 2). Figure 2 reveals a significant positive change (increasing use) in both total antibiotics (*p* = 0.0021) and restricted antibiotic cost/patient/day (*p* = 0.0037) due to the deconstruction of the stewardship team (interruption #1–1st solid vertical line). However, once reconstruction of the team occurred (interruption #2–2nd solid vertical line), a significant negative change (*p* ≤ 0.001, respectively for both) was observed (R^2^ = 0.51 and 0.45, respectively).

A significant increase in cost for both overall total antibiotics (*p* = 0.0039) and total antibiotics per day (*p* = 0.01) was also observed related to the deconstruction of the team. However, when reconstruction of the team occurred, a significant decrease in cost (*p* < 0.0001 for both) was observed (R^2^ = 0.34, 0.30, respectively). 

Further, a significant positive change in percent restricted to total antibiotics (*p* = 0.0135) was observed related to deconstruction. Once the reconstruction occurred, a significant negative change (*p* = 0.0033) was observed (R^2^ = 0.58).

## 4. Discussion

In 1998, we initiated our hospital wide effort at antibiotic stewardship. Elements of our initial program aligned well with the position adopted by the IDSA with regard to antibiotic stewardship goals and daily practice elements. 

On a monthly, quarterly, and yearly basis, we monitored overall antibiotic purchases (Figure 1), the percent of the inpatient pharmaceutical budget made up by antibiotics, overall antibiotic cost per patient per day (Figure 1), restricted antibiotic cost per patient per day, and the percent of our antibiotic dollar represented by restricted antibiotics. As we could not benchmark our performance/results against other similar institutions in 1998, we made a decision to compare ourselves to ourselves over time. While the sensitivity of antibiotic purchase dollars is not an ideal metric today, the comparison is tempered by the longitudinal period of time involved in our internal comparison. In retrospect, use of defined daily doses or antibiotic days of therapy may have provided a better reflection of antibiotic consumption. However, in 1998, the readily available and retrievable parameter for our purpose was dollars expended. 

Another expected lesson over the 14-year experience is that a program of antibiotic stewardship is likely to change over time. Pharmacy and medical personnel will change and the various tools that can be used to affect prescriber behavior may need to be rotated into and out of the stewardship program. Further, some elements, such as our trial of the antibiotic order form, did not work for us and failed to have the desired impact in our system. Stewardship programs over time are also likely to experience a fatigue factor especially in a teaching institution as prescribers rotate in and out throughout the year. Likely each institution will have to find the right stewardship niche that works for them and that may change somewhat from year to year. Lastly, while these programs are attractive, barriers often exist to procuring enlisting the specified personnel identified in the IDSA position paper and the provision of financial support for dedicated time on task [2].

The IDSA primary goal for antibiotic stewardship is to optimize antibiotic clinical outcomes while minimizing unintentional consequences of antibiotic therapy [2]. We have no definitive data that suggesting that that goal was realized in our program. However, in regard to the secondary goal of reducing healthcare costs, this study demonstrates a statistically significant alteration in antibiotic costs when the antibiotic stewardship effort is interrupted and then restarted. (Figure 2) 

Most ASPs can demonstrate an initial reduction in antibiotic costs. In this report we demonstrate the initial financial effect of our ASP, the increasing costs associated with the interruption of the program, and the return of financial stability when the program was restarted. These unique circumstances lent themselves to a segmented time sequence series analysis, which provided statistical evidence of the ASP effect on antibiotic costs.

## 5. Conclusions

We demonstrate the need for the development and continued support of an ASP. Antibiotic costs were significantly influenced by the presence or absence of a functioning ASP during 14 years of operation/observation at Regions Hospital. The successes reported here should encourage hospitals and health-care providers to initiate and continue ASPs.
